# A Rapid and Sensitive HPLC-Fluorescence Method for Determination of Mirtazapine and Its two Major Metabolites in Human Plasma

**Published:** 2014

**Authors:** Hoda Lavasani, Mario Giorgi, Behjat Sheikholeslami, Mohammadhasan Hedayati, Mohammad Reza Rouini

**Affiliations:** a *Biopharmaceutics and Pharmacokinetic Division, Department of Pharmaceutics, Faculty of Pharmacy, Tehran University of Medical Sciences, Tehran, Iran.*; b *Department of Veterinary Sciences, University of Pisa, Via Livornese (Lato Monte) 1, San Piero a Grado 56010 Pisa, Italy. *; c *Chemical and Petroleum Engineering Department, Sharif University of Technology. *; d *Pharmaceutical Quality Assurance Research Centre, Faculty of Pharmacy, Tehran University of Medical Sciences, Tehran, Iran. *

**Keywords:** Mirtazapine, Metabolites, HPLC, Plasma, Fluorescence

## Abstract

A rapid and sensitive HPLC method has been developed for the quantification of mirtazapine (MRZ), a noradrenergic and specific serotonergic inhibitor antidepressant (NaSSA) and its two major metabolites N-desmethyl mirtazapine (NDM) and 8-hydroxymirtazapine (8-OHM) in human plasma.

The separation was achieved using Chromolith C_18 _column and a mobile phase of acetonitrile: phosphate buffer (pH = 3, 20:80, v/v) in isocratic mode at a flow rate of 2 mL/min. A fluorescence detector was set at 290 and 350 nm for excitation and emission, respectively. Zolpidem was used as the internal standard. Liquid-liquid extraction was applied for sample clean up. All analytes were eluted in less than 5 minutes with LOQ of 1 ng/mL for MRZ and 2 ng/mL for both NDM and 8-OHM. The developed method was successfully applied to quantify MRZ and its metabolites in plasma of a healthy volunteer.

## Introduction

Mirtazapine (MRZ) has a piperazinoazepine structure {1,2,3,4,10,14b-hexahydro-2- methylpyrazino [2,1-a]-pyrido [2,3-c][2] benzazepine}(1). It belongs to the class of noradrenergic and specific serotonergic inhibitor antidepressants (NaSSA) (2). MRZ increases the central noradrenergic and serotonergic activity by blocking α2 adrenergic receptors. MRZ also acts as an antagonist of postsynaptic serotonin type 2 (5-HT2) and type 3 (5-HT3) (2). The drug has a different mechanism compared to most of the second generation antidepressants, and it is used to treat generalized anxiety (3), obsessive–compulsive (4) and post traumatic stress disorders (5). It has a high affinity for histamine H1 receptors; which consequently produces sedation following administration (2). 

MRZ is bio-transformed in the liver by the isoforms 2D6, 3A4 and 1A2 of cytochrome P450 (CYP) (6, 7). Several metabolites of MRZ have been isolated from human liver microsomes and rat hepatocytes (8-10). Demethylation and oxidation are the two main metabolic pathways for MRZ (11). The urinary excretion profile reported in humans demonstrated that 25% of MRZ is N-demethylated by CYP3A4 to desmethyl mirtazapine (NDM) and 40% is oxidated by CYP2D6/CYP1A2 to 8 hydroxy mirtazapine, (8-OHM) (12). NDM (also called nordesmethylmirtazapine) is the major metabolite. It is pharmacologically active with a potency of about 15% as compared to the parental drug (13). 

MRZ is a basic, lipophilic drug with a pKa value 7.1 (14). Its binding to plasma proteins is 85% (15) and it has a bioavailability of almost 50% after a single oral dose in humans (16). 

Several high-performance liquid chromatographic (HPLC) methods with ultraviolet (17-19), fluorescence (20-25) and mass (26-28, 30) detectors have been developed for the determination of MRZ and NDM in plasma. 

The major drawback of the previously described protocols is the labour intensive liquid–liquid extraction as a sample preparation method. Moreover, in most published methods only MRZ and NDM have been quantified. The method reported by Mandrioli *et al. *(21) quantified MRZ and both the major metabolites but SPE were used to purify the samples. This led to a protracted analytical process and the reported LOQS for the analytes were not suitable for a pharmacokinetic study. 

Fernando Jose *et al*. (27) have also determined MRT, NDM and 8-OHM using a mass detector with a rapid sample purification resulting in a good LOQ. However, the drawback of this method is that the LC/MS device is still not widely available in laboratories. Hence, the aim of the present study is to develop a rapid HPLC method coupled with a fluorescence detector to quantify MRZ and its two main metabolites in human plasma within a reasonable time, thus retaining adequate sensitivity for a pharmacokinetic study. 

## Experimental

*Chemicals and reagents*

**Figure 1:**
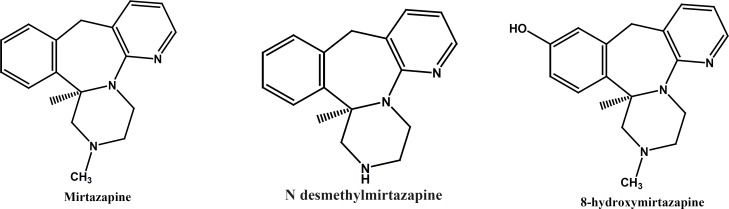
Chemical structure of mirtazapine and its major metabolites

The pure substances of MRZ, NDM and 8-OHM (Figure 1) were kindly supplied by Organon (Oss, Netherlands). Zolpidem, the internal standard was generously gifted by Osvah pharmaceutical co. (Tehran, Iran). HPLC-grade acetonitrile and methanol and analytical grade ethyl acetate, *n*-hexane, phosphoric acid (85%) and potassium dihydrogen phosphate were supplied by Merck (Darmstadt, Germany). 

*Apparatus and chromatographic condition*

The chromatographic apparatus consisted of a low-pressure gradient HPLC pump, a fluorescence detector (excitation wavelength (λex) 290 nm/emission wavelength (λem) 370 nm) and an online degasser, all from Knauer (Berlin, Germany). A Rheodyne model 7725i injector with a 100 μL loop was used. 

The data was acquired and processed using Chrom Gate chromatography software (Knauer). Separation was achieved by Chromolith^TM^ Performance RP-18e 100 mm×4.6 mm column (Merck, Darmastadt, Germany) protected by a Chromolith^TM^ Guard Cartridge RP-18e 5 mm×4.6 mm. Mobile phase consisted of acetonitrile:0.025 M monobasic potassium phosphate buffer adjusted to pH 3 by phosphoric acid (20:80, v/v) and was delivered in an isocratic mode at a 2 mL/min flow rate.

*Preparation of standard solutions*

Stock solutions of MRZ, NDM and 8-OHM were prepared separately in methanol to achieve a concentration of 1 mg/mL. Intermediate stock standards of 100 and 10 μg/mL were prepared in double distilled water. Three standard solutions of MRZ and 8-OHM (10, 50 and 100 ng/mL) and NDM (20, 100 and 200 ng/mL) were made by further dilution of the intermediate stock solution with appropriate volumes of water. A standard solution of the internal standard (Zolpidem) was titrated to a concentration of 1 mg/mLusing methanol. Standard and stock solutions of all compounds were stored at 4 °C until use.

*Preparation of calibration standards*

A calibration curve comprising 12 points was generated, this covered the concentration ranges of 1-500 ng/mL for MRZ and 8-OHM and 2-500 ng/mL for NDM. These concentrations were prepared by diluting the pooled aqueous stock solutions of 10 μg/mL for MRZ and 8-OHM and 20 μg/mL for NDM with drug free human plasma obtained from healthy volunteers.

**Table 1:** Limit of quantitation (LOQ) for MRZ, NDM and 8-OHM (n=5).

**Analyte **	**Conc (ng/mL) **	**Between-day**		**Within-day**			
		**Precision**	**Accuracy**	**Precision**		**Accuracy**
MRZ	1	11.2	112.8	12.9	118.2		
NDM	2	18.8	117.0	13.9	98.1		
8-OHM	2	13.1	98.4	19.7	107.9		

*Sample preparation*

A 150 μL aliquot of plasma was transferred into a 2 mL Eppendorf polypropylene tube; 50 μL Zolpidem as an internal standard (7.5 ng/mL) and 50 μL NaOH (1 N) were then added and mixed. The mixture was extracted with 1.5 mL of *n*-hexane:ethylacetate (90:10 v:v). After vertical agitation (15 min) and centrifugation (10,000×g, 10 min), the supernatant was collected in a 10 mL conical glass tube. The organic phase was then evaporated under a gentle stream of air and reconstituted in 150 μL of mobile phase. A 100μL aliquot was finally injected in to the HPLC system.

*Analytical method validation*

The present method was validated throughevaluation of selectivity, specificity, linearity, the limit of quantification (LOQ), precision, accuracy, extraction recovery and stability according to U.S. Food and Drug Administration (FDA) guidelines (30).

*Selectivity*

The selectivity of the method was investigated by analyzing six individual human blank plasma samples in order to detect any endogenous plasma compounds that may interfere with the analytes and I.S.

*Linearity*

Under the described HPLC method, the calibration curve was generated by a least squares linear regression of peak-area ratios of analytes to the I.S. versus respective plasma concentrations over the range of 1-500 ng/mL for MRZ and 8-OHM and 2-500 ng/mL for NDM. 

*Limit of quantitation*

The LOQ was defined as the lowest analyte concentration that could be identifiable with an accuracy of 80-120% and a precision below 20%. In the present method, the lowest concentration on the standard curve was considered as the limit of quantitation (LOQ).

*Accuracy and precision*

Accuracy and between- and within-day precision of the method were determined for each compound. Five replicate spiked plasma samples were assayed between-days and within in single day at three different concentrations (10, 50 and 300 ng/mL) for all analytes. Accuracy was calculated as deviation of the mean from the nominal concentration. Between- and within day precision was expressed as the relative standard deviation of each calculated concentration.

*Extraction recovery*

Average recovery of each compound was determined by comparing the peak area obtained after injection of the replicate processed quality control (QC) samples (10, 100 and 500 ng/mL) with those achieved by direct injection of the same amount of analyte in standard solution in mobile phase (n=5)

*Stability*

The analytes’ stability in stock solutions and in QC plasma samples (10, 100 and 500 ng/mL) under various conditions was evaluated. Room temperature stability of stock solutions was assessed in 1, 2 and 4 weeks. Freeze-thaw stability test was carried out after performing five repeated freeze-thaw cycles on three QC samples. The long-term stability was analyzed using QC samples kept at -20 °C for 1, 2 and 6 months.

*Method application*

The validated method was applied to define the pharmacokinetic parameters of MRZ and its main metabolites in a healthy volunteer after oral administration of 45mg of MRZ (45 mg tablet, Mirtazapine Sandoz, Sandoz). The protocol was approved by the Ethics Committee of the Faculty of Pharmacy, Tehran University of Medical Sciences and written informed consent was obtained from a volunteer who participated in the study before blood sampling. Blood samples were collected into a heparinized tube at zero, 0.25, 0.5, 0.75, 1, 1.5, 2, 3, 4, 5, 6, 8, 10, 24 and 48 hours after dosing. Plasma was separated by centrifugation at 1850 × g for 15 min and was stored at -20 °C until analysis. Non-compartmental pharmacokinetic evaluation was carried out using the WinNonlin 5.3 program (Pharsight) .

## Results and Discussion

*Method development*

**Figure 2:**
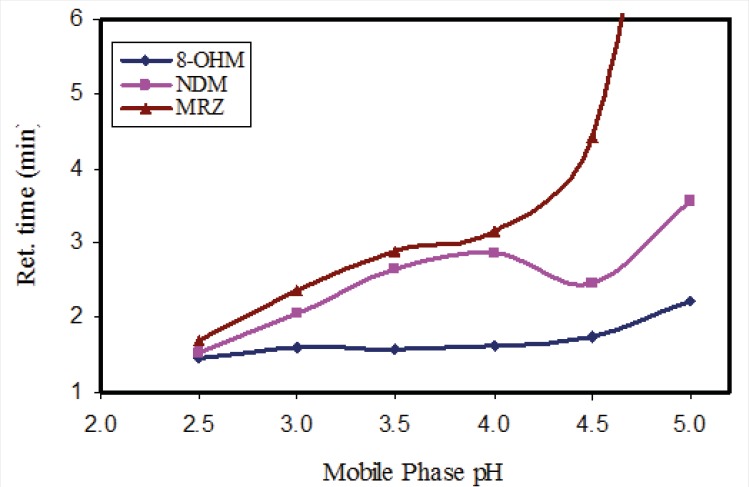
Effect of the pH of mobile phase on the retention time of MRZ, NDM and 8-OHM

In order to evaluate the influence of pH of the mobile phase, phosphate buffer solutions with different pH values were tested. While the resolution and retention time of 8-OHM slightly increased in parallel to incremental increases in pH, a significant effect on the retention times of MRZ and NDM was observed with increases in pH. With a pH higher than 4.5, a drastic increase in retention time and peak width of MRZ was observed (Figure 2). A pH of 3 was finally selected because of suitable resolution and retention times (almost 4 minutes) of all analytes. It was also noteworthy that with any increase in pH of the mobile phase, the peak intensity of all three analytes decreased (almost 15% decrease in peak intensity from pH 2.5 to 5 was observed). In addition, both the recovery and florescence intensity were also influenced by pH of the reconstituting solution.

The use of methanol as the organic solvent instead of acetonitrile in the mobile phase caused an overlap between the MRZ and NDM peaks. Any combination of methanol and acetonitrile did not enhance this resolution. Therefore, acetonitrile was selected as the organic modifier because it resulted in lower back pressure and good resolution between peaks. 

To increase the separation efficiency required for resolution of all compounds, two Chromolith C_18 _columns were connected in line to produce a column with high plate count (10.000 typical plate numbers with one column and 19.000 with two columns) at relatively low back-pressure.

Several compounds were tested as internal standards. Some of tested drugs did not show acceptable fluorescence intensity and some did not have suitable retention times. Zolpidem was eventually selected as the internal standard. Zolpidem showed very good peak intensity at very low concentrations. Its recovery on extraction was both acceptable and reproducible.

*Sample preparation*

Requirements for an effective extraction method include providing accurate quantitative extraction of MRZ and its metabolites in plasma and minimizing interference from the matrix. Protein precipitation using methanol or other organic solvents and a variety of precipitating salts caused interference with some plasma components and decreased the LOQ and selectivity of the method. 

**Figure 3:**
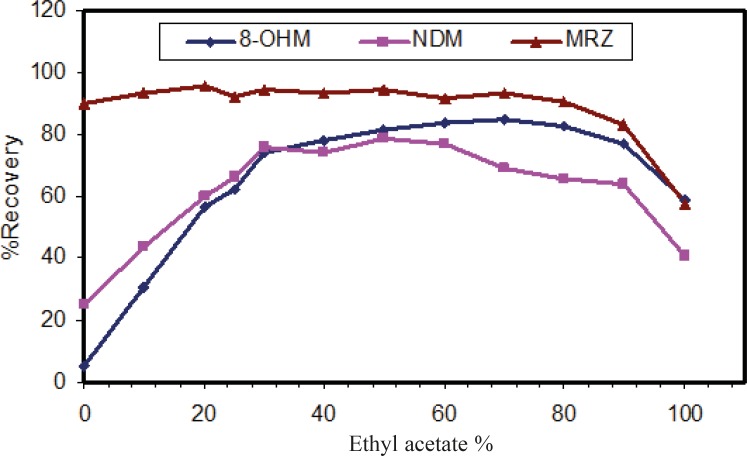
Effect of organic solution mixture on recovery of analytes

**Figure 4:**
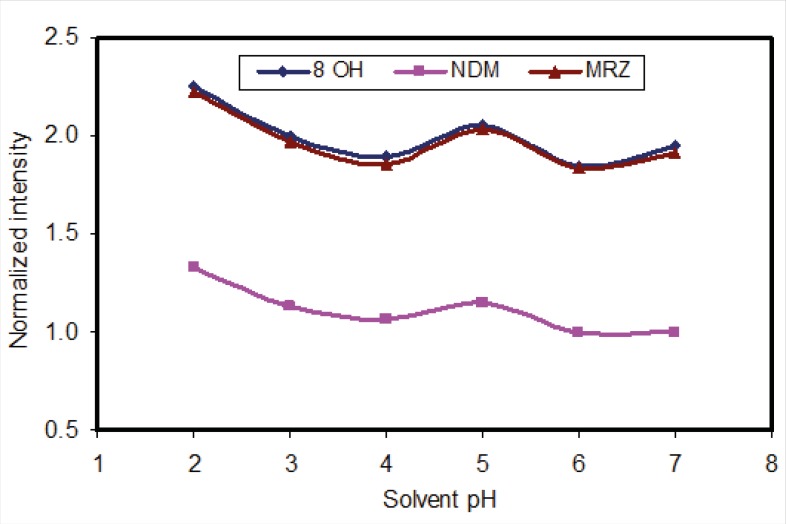
Effect of pH of constituent solvent, on the fluorescence intensity of analytes (all intensities were normalised to intensity of NDM at pH=7).

The liquid-liquid extraction method was examined with several solvent types. Different organic solvents such as ethyl acetate, n-hexane and toluene were assessed. Extraction with toluene produced additional peaks in close proximity to those corresponding to the retention time of 8-OHM. However, using *n*-hexane and ethyl acetate, these interferences disappeared. Extraction with pure *n-*hexane (zero percent ethyl acetate) enhanced the recovery of NDM but resulted in decreased 8-OHM recovery. Using a mixture of n-hexane and ethyl acetate resulted in more efficient extraction of 8-OHM from the matrix; the optimal ratio of these solvents in terms of extraction efficiency, selectivity and reproducible response was evaluated and finally a composition of *n*-hexane/ethyl acetate (90/10, v/v) was selected (Figure 3). It should be mentioned that as for mobile phase pH, peak intensity of extracted analytes was dependent on pH of reconstituting solvent (Figure 4). Hence, mobile phase was selected as the reconstituting solvent. 

*Selectivity and specificity*

**Figure 5:**
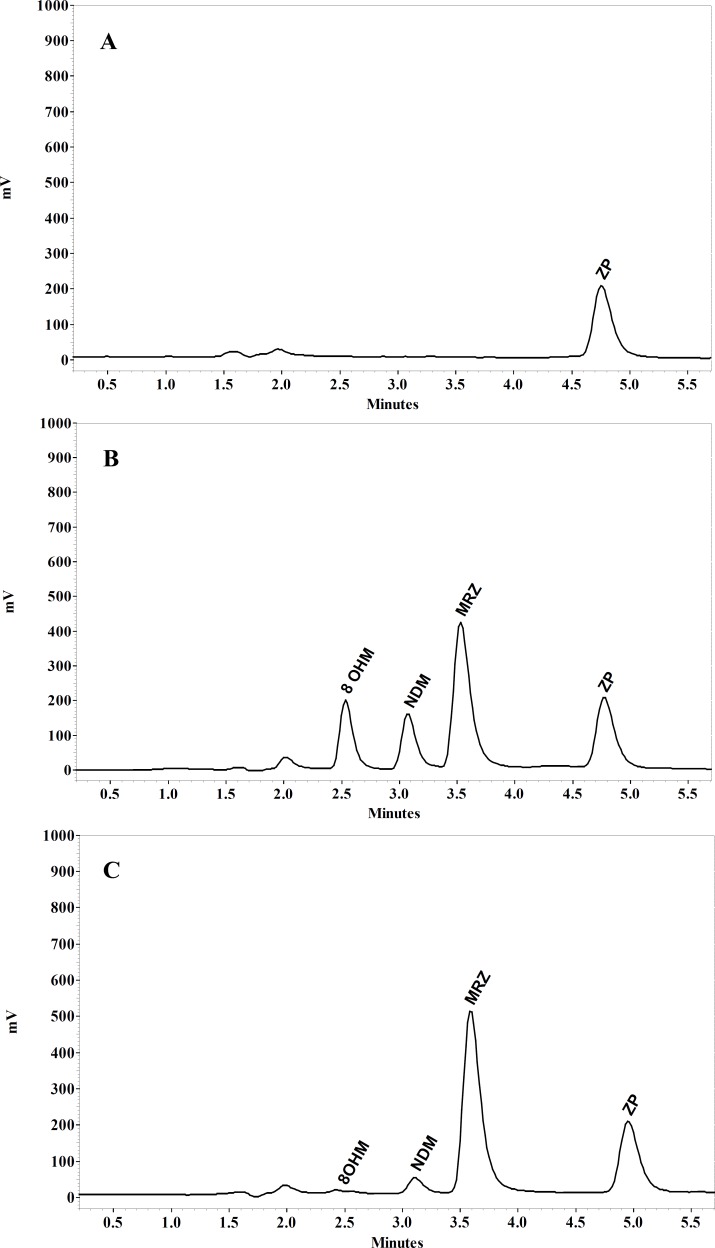
Chromatograms of (A) blank human plasma spiked with IS, (B) plasma spiked 100 ng/mL of MRZ and 8-OHM and 50 ng/mL NDM and (C) plasma of the volunteer 2.5 h after single oral dose of 45 mg MRZ

MRZ and its two main metabolites separated by conditions described in the present study are demonstrated in Figure 5. Retention times of MRZ, NDM, 8-OHM and Zolpidem (IS) were 3.5, 3, 2.6 and 4.9 min respectively. No endogenous interference with analytes and I.S. was observed in the different sources of blank plasma.

To prove the selectivity of the developed chromatographic method, separation was performed in the presence of several structurally similar antidepressant compounds and there was no interference at the retention time of all analytes.

*Limit of quantitation (LOQ)*

LOQ was determined as the lowest concentration which had between 80-120% accuracy and below 20% precision. LOQ was 1 ng/mL for MRZ and 2 ng/mL for the metabolites (Table 2).

**Table 2:** Between- and within-day variability, accuracy, and recovery for determination of MRZ, NDM and 8-OHM (n=5).

**Conc.(ng/mL)**	**Between-day**		**Within-day **			**Recovery **	
	**Precision**	**Accuracy **	**Precision**	**Accuracy **	**(%)**		**SD**
MRZ							
5	5.50	103.14	12.9	118.3	82.5		9.6
100	4.07	95.25	2.23	99.8	88.6		8.5
500	7.80	101.33	5.03	90.02	89.5		8.9
NDM							
10	12.88	106.8	16.7	81.1	32.3		2.5
100	11.87	96.8	9.8	82.6	37.5		3.5
500	8.60	88.6	11.3	81.2	41.9		2.1
8-OHM							
5	17.6	118.5	3.9	106.6	24.5		2.6
100	14.81	101.6	1.7	105.1	23.6		2.1
500	11.15	102.9	2.9	113.3	28.0		2.9

**Figure 6:**
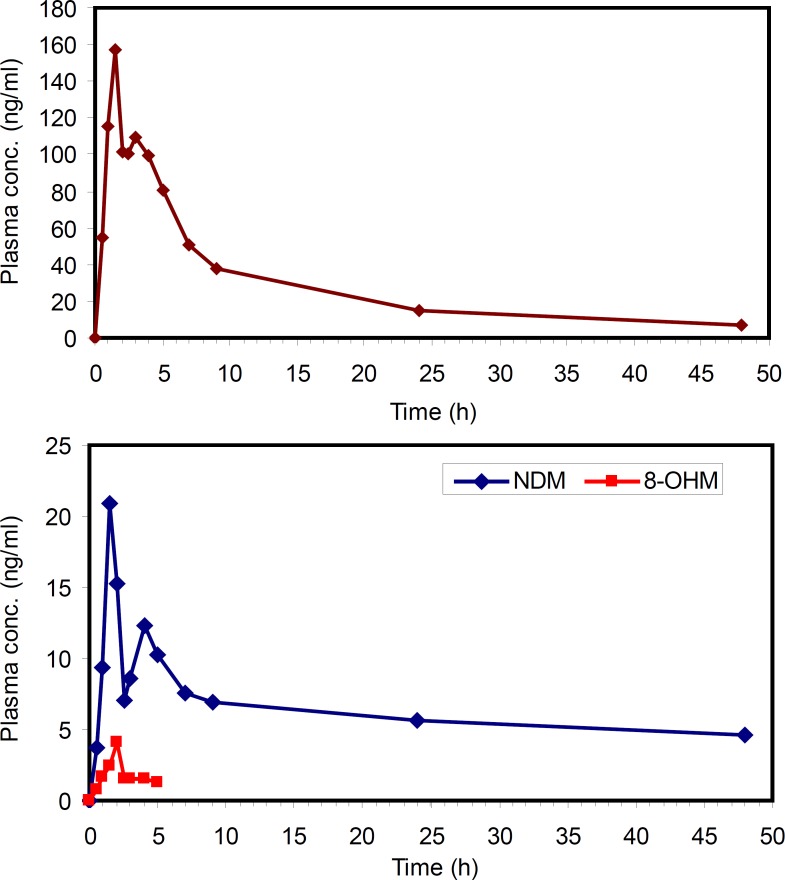
Concentration-time profiles of MRZ (Upper panel) and its metabolites (Lower panel) after oral administration of 45 mg mirtazapine tablet to a healthy volunteer

*Linearity*

Linearity of the method was assessed over the concentration range of 1–500 ng/mL for MRZ and 8-OHM and 2–500 ng/mL for NDM. There was a linear relationship between peak area ratios and related concentrations when linear regression analysis was applied. The regression equation for each analyteis as follows: 

MRZ; y = 0.034x + 0.0024 (r2 = 0.997) 

NDM; y = 0.0058x - 0.0301 (r2 = 0.991) 

8-OHM; y = 0.011x - 0.0033 (r2 = 0.999). 

*Accuracy, precision and extraction recovery*

Accuracy at low, medium and high concentration levels (10, 100 and 500 ng/mL) ranged from 81.1% to 118.5% for all compounds. The within- and between-day precision indicated the reproducibility of the method ranged from 1.7% to 17.6% for the three analytes. The mean extraction recovery was 86.8%, 37.2% and 25.3% for MRZ, NDM and 8-OHM, respectively.

The recovery of MRZ using our extraction method is comparable to previously published methods. However, the data regarding NDM is somewhat different. We could not attain a recovery higher than 30% for this metabolite. Although, with this low recovery, the LOQ of NDM was low enough to detect its plasma concentrations for 48 h in the healthy volunteer. Maris *et al. *(23) have reported a recovery of about 40% for NDM after double extraction with *n*-hexan. Romiguieres *et al*. (17), reported a recovery of about 100% but with a LOQ of only 20 ng/mL. Ptacek *et al. *(20) have also developed a sensitive HPLC method with a LOQ of 1.5 ng/mL for NDM but its recovery was not reported.

As far as the 8-OHM recovery is concerned, it was around 30%. The only non-MS HPLC method which has quantified this metabolite used solid phase extraction with a long washing process of SPE cartridge and evaporation of methanol in a rotary evaporator (21). Using this method a recovery of almost 97% was achieved. That is a much higher yield than the recovery value for 8-OHM obtained in the present study.

*Stability*

No significant decrease was observed in the concentration of MRZ and its metabolites in three different conditions (at room temperature for 4 weeks, -20 °C for 6 months and after five repeated freeze-thaw cycles). The entire relative standard deviation was less than 5%.

*Application of the method*

The applicability of this method was verified by determining MRZ and its metabolites in human plasma after oral administration of a 45 mg single dose of MRZ to a healthy volunteer. The concentration of MRZ in plasma reached a Cmax of 157 ng/mL at 1.5 hours after drug administration. The area under the plasma concentration-time curve of MRZ from zero to 48 hours AUC(0-48) was calculated to be 1360 ng.h/mL. A half life of 14.5 hours was calculated from log-linear regression of 5 last terminal points. At 1.5 hours post administration, NDM reached a maximum of 19 ng/mL and thereafter concentration decreased with a half life of around 10 hours. The AUC (0-48) for this metabolite was calculated to be 301 ngh/mL. The 8-OHM was only detectable up to 5 hours after drug administration with a maximum concentration of 4.5 ng/mL. This method allowed quantification of MRZ and NDM for 48 h after administration of MRZ at the lowest clinical dose rate. That is critical because MRZ has recently been reconsidered in the treatment of various states such as pain, anorexia and emesis, in both humans and veterinary medicine (31-33).This method could be successfully applied in the PK evaluation of the above mentioned cases.

*Conflict of interest statement*

None of the authors of this paper has a financial or personal relationship with other people or organisations that could inappropriately influence or bias the content of the paper.
